# Glucocorticoid receptor action in prostate cancer: the role of transcription factor crosstalk

**DOI:** 10.3389/fendo.2024.1437179

**Published:** 2024-07-04

**Authors:** Johannes Hiltunen, Laura Helminen, Ville Paakinaho

**Affiliations:** Institute of Biomedicine, University of Eastern Finland, Kuopio, Finland

**Keywords:** androgen receptor, chromatin, crosstalk, glucocorticoid receptor, prostate cancer, transcription factor

## Abstract

Prostate cancer is one of the most prevalent malignancies and is primarily driven by aberrant androgen receptor (AR) signaling. While AR-targeted therapies form the cornerstone of prostate cancer treatment, they often inadvertently activate compensatory pathways, leading to therapy resistance. This resistance is frequently mediated through changes in transcription factor (TF) crosstalk, reshaping gene regulatory programs and ultimately weakening treatment efficacy. Consequently, investigating TF interactions has become crucial for understanding the mechanisms driving therapy-resistant cancers. Recent evidence has highlighted the crosstalk between the glucocorticoid receptor (GR) and AR, demonstrating that GR can induce prostate cancer therapy resistance by replacing the inactivated AR, thereby becoming a driver of the disease. In addition to this oncogenic role, GR has also been shown to act as a tumor suppressor in prostate cancer. Owing to this dual role and the widespread use of glucocorticoids as adjuvant therapy, it is essential to understand GR’s actions across different stages of prostate cancer development. In this review, we explore the current knowledge of GR in prostate cancer, with a specific focus on its crosstalk with other TFs. GR can directly and indirectly interact with a variety of TFs, and these interactions vary significantly depending on the type of prostate cancer cells. By highlighting these crosstalk interactions, we aim to provide insights that can guide the research and development of new GR-targeted therapies to mitigate its harmful effects in prostate cancer.

## 1 Introduction

The glucocorticoid receptor (GR) is a member of the steroid receptor (SR) family, which belongs to the nuclear receptor superfamily (1, 2). Specifically, GR (NR3C1) is classified as one of the 3-ketosteroid receptors (NR3Cs), along with the mineralocorticoid receptor (MR, NR3C2), progesterone receptor (PR, NR3C3), and androgen receptor (AR, NR3C4). These NR3C receptors share a highly conserved DNA-binding domain, resulting in similarly conserved hormone responsive elements (HREs) for the corresponding receptors. The estrogen receptor (ER), a related SR, has a different DNA binding motif from other NR3Cs due to structural differences in its DNA-binding domain. Typically, but not always, unliganded SRs reside in the cytoplasm, bound to heat shock protein complexes. Upon ligand binding to the receptor’s ligand-binding domain, they are translocated to the nucleus (1, 3). The common natural ligands for SRs include cortisol for GR, aldosterone for MR, progesterone for PR, dihydrotestosterone (DHT) for AR, and estradiol for ER. Once in the nucleus, SRs oligomerize and bind to regulatory elements at enhancers, thereby achieving transcriptional regulatory capabilities. However, the exact nature of oligomerization is still debated, with evidence suggesting that SRs can form tetramers (4, 5). Moreover, the physiological relevance of GR monomer, which has long been thought to drive the beneficial anti-inflammatory effects of GR, is now being challenged, as monomeric GR is practically nonfunctional (6, 7). Once SRs have formed oligomers and bound to chromatin, they recruit coregulators and other transcription factors (TFs) to the site. These recruited proteins often possess enzymatic activities required to modulate chromatin accessibility, enhancer activity, and RNA polymerase activity. Despite subtle differences, all SRs function in a similar fashion to regulate gene expression.

Expressed in nearly every human tissue, GR is associated with several indispensable pathways, including metabolism, development, stress response, and inflammation (8). Consequently, GR is considered a desirable therapeutic target for controlling inflammation (9). However, long-term glucocorticoid usage can lead to a plethora of detrimental side effects (10), such as osteoporosis, diabetes, and cardiovascular diseases. These adverse effects often arise from GR-mediated changes in glucose metabolism, which limits the utility of glucocorticoids as a long-term therapy. Prostate cancer, one of the most common cancers and leading causes of cancer-related deaths in men in Finland and worldwide (11–13), is often treated with glucocorticoids. In the management of prostate cancer patients, glucocorticoids are primarily administered to mitigate the side effects of chemotherapy and to alleviate inflammation (14).

Despite the widespread use of glucocorticoids, our understanding of the functionality of GR in prostate cancer remains limited, particularly across different subtypes of the disease. Early investigations suggested that glucocorticoids may inhibit prostate cancer tumor growth by restricting the activity of oncogenic TFs and mitogen-activated protein kinase (MAPK) signaling pathways (15). However, glucocorticoids can also confer oncogenic properties in certain contexts, as evidenced by therapy resistance emerging from GR-mediated maintenance of AR signaling during antiandrogen treatments (16). Given GR’s dual role in prostate cancer—either as a tumor suppressor or an oncogenic TF—it is crucial to understand the factors that drive its functional direction. One potential explanation lies in GR’s interactions with other TFs through various crosstalk mechanisms. Indeed, many critical aspects of GR functionality are intertwined with its crosstalk with other TFs (17). Therefore, deciphering the crosstalk mechanisms underlying GR action in prostate cancer could pave the way for the discovery of new therapeutic targets. The importance of developing novel approaches to target GR signaling is underscored by recent clinical trials showing no significant clinical benefit when combining AR and GR inhibition (18, 19). Thus, in this review, we aim to elucidate the diverse crosstalk mechanisms involving GR in prostate cancer.

## 2 Transcription factor crosstalk mechanisms of GR

**Figure 1:**
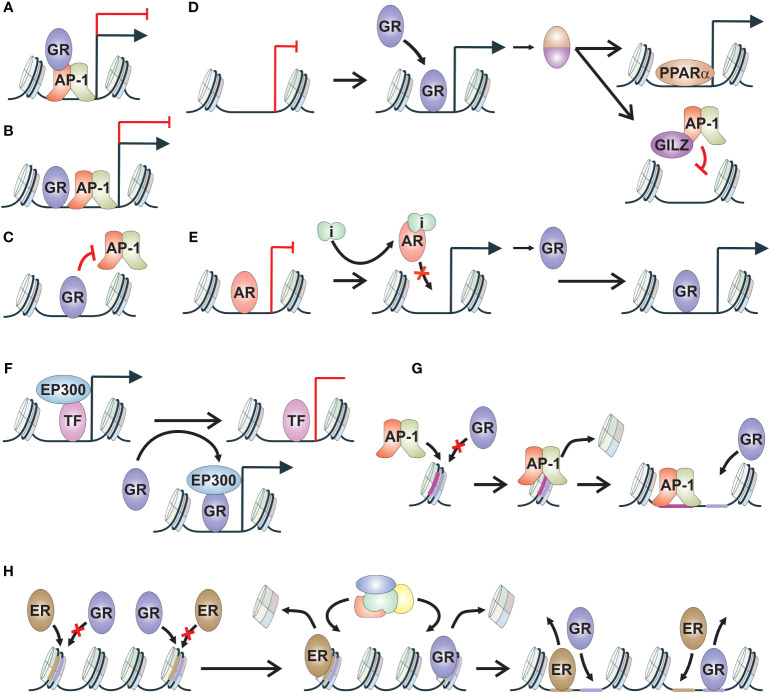
Mechanisms of GR crosstalk. **(A)** Tethering. TF, such as GR, tethers via protein-protein contacts to another TF, such as AP-1, modulating its transcriptional activity. **(B)** Composite binding. TFs, such as GR and AP-1, bind to adjacent sites on chromatin and modulate transcriptional regulation. **(C)** Direct blocking. TF, such as GR, binds to motif of another TF, such as AP-1, thereby inhibiting its chromatin binding. **(D)** TF cascade. TF, such as GR, induces the gene expression of a secondary protein, such as PPARα or GILZ. These proteins can regulate their canonical target genes, such as PPARα, or block the transcriptional activity of additional TF, such as AP-1 blocked by GILZ. **(E)** Reverse TF cascade. TF, such as AR, represses the gene regulation of a secondary protein, such as GR. Inhibition of TF activity leads to increased production of secondary protein and in the regulation of its target genes. **(F)** Coregulator squelching. Activation of a TF, such as GR, leads to sequestration of coregulator, such as EP300, from the enhancer of another TF, leading to alterations in gene regulation. **(G)** Pioneer factor. TF, such as AP-1, induces chromatin binding of secondary TF, such as GR, through chromatin remodeling. Secondary TF is unable to bind to the closed chromatin site without the activity of the pioneer factor. **(H)** Assisted loading. Initiator TF, such as GR, induces chromatin binding of secondary TF, such as ER, through chromatin remodeling. At other sites, the role of initiator and secondary TF is switched. AP-1, activator protein 1; AR, androgen receptor; ER, estrogen receptor; GILZ, glucocorticoid-induced leucine zipper; GR, glucocorticoid receptor; i, inhibitor; PPARα, peroxisome proliferator-activated receptor alpha; TF, transcription factor.

Upon exposure to ligands, GR becomes activated and translocates to the nucleus, where it interacts with chromatin, other TFs and coregulators to regulate numerous physiological pathways. Due to its ligand-dependent nature, like many other nuclear receptors, GR has been extensively utilized to elucidate a variety of TF crosstalk mechanisms (20, 21). GR has been observed and postulated to exert its effects through multiple crosstalk mechanisms, influencing the transcriptional activities of other TFs and vice versa (**Figure 1**). With its widespread expression across tissues, GR’s crosstalk with other TFs ultimately determines the receptor’s context- and tissue-specific effects (2, 17, 22). This is exemplified by the dependence of GR binding site locations in the target tissue (17, 23, 24). GR’s interactions with tissue-specific TFs are postulated to modulate chromatin accessibility and GR binding to these sites (23). Furthermore, cell type-specific GR binding sites often lack HREs, exhibit an open chromatin configuration, and are co-occupied by other TFs (25). These findings underscore the role of tissue-specific TFs in fine-tuning the action of GR. However, GR itself can, to some extent, modulate the binding of tissue-specific TFs (26, 27).

Many early insights into GR crosstalk mechanisms stemmed from the well-known anti-inflammatory effects of glucocorticoids. These anti-inflammatory capabilities are mediated by GR through the inhibition of inflammatory and immune-related signaling pathways regulated by several TFs. Among the most notable TFs are activator protein 1 (AP-1), nuclear factor kappa-light-chain-enhancer of activated B cells (NF-κB), and signal transducer and activator of transcription (STAT) (28–31). GR employs various mechanisms to modulate the activity of these TFs (32, 33).

Due to the abundance of GR binding sites lacking canonical HRE, termed glucocorticoid response element (GRE), protein-protein interactions, particularly tethering of GR to inflammatory TFs, have emerged as central mechanisms for its anti-inflammatory functions (**Figure 1A**) (34, 35). Tethering involves the recruitment of TF, such as GR, to a DNA bound TF, such as NF-κB, resulting in the activation or repression of transcription (36, 37). The repressive tethering between GR and inflammatory TFs can be mutual. For instance, the p65 subunit of NF-κB has been shown to reciprocally modulate GR activation (36, 37). In macrophages, GR-mediated repression of NF-κB and AP-1 target gene expression is mediated by NCOA2 (GRIP1) (38). NCOA2 is a common coactivator recruited to chromatin by GR to induce the expression of its target genes. Thus, a classical SR coactivator can mediate context-specific corepressive functions through GR-mediated tethering. Intriguingly, phosphorylation of NCOA2 has been identified as a fine-tuning mechanism for its coactivator-versus-corepressor activity (39). In addition to NCOA2, other coregulators are involved in modulating GR’s anti-inflammatory activity, such as SETD1A and BRD9 (40, 41). Finally, tethering can modulate inflammatory TF-regulated transcription in a context-dependent manner. It was observed that in STAT3-GR tethering, GR tethering to STAT3 inhibited transcriptional activity, while STAT3 tethering to GR increased transcriptional activity (29).

When GREs overlap or are closely associated with inflammatory TF motifs, they are thought to act through the so-called composite binding sites (**Figure 1B**) (42). Composite motifs involving GR and AP-1 have been established since the 1990s, with the composition of AP-1 subunits correlating with negative or positive regulation of GR action (43). Furthermore, composite binding has been observed between GR and STAT3, where the binding of both TFs was necessary for stabilizing the chromatin loading of the other (29). Additionally, a composite motif comprising both the STAT3 binding element and GRE was found to induce synergistic gene activation. Although physical interactions are often presumed to occur, some interactions can possibly occur through conformational changes in DNA, potentiating the interactive effects (44). Composite binding activity of GR resembles cooperative TF binding, involving combinatorial control of gene expression, where the action of at least two TFs is needed to gain access to binding sites in closed chromatin regions (20, 44). However, this type of GR crosstalk has not been extensively investigated.

Recently, it has been suggested that for most GR-mediated repression of inflammation signaling, direct DNA binding of the receptor is required (**Figure 1C**). In macrophages stimulated with inflammatory lipopolysaccharides, only one fifth of GR-mediated repression was determined to be related to tethering events, with the majority being regulated by direct DNA binding of GR (45). A study with zinc finger point-mutated GR unable to bind DNA, provided evidence that even though tethering is maintained, direct DNA binding is necessary to assemble coregulator complexes and convey transcriptional repression through GR (46). Additionally, direct DNA binding of GR has been shown to occur at NF-κB and AP-1 response elements that mediate inflammatory response (47, 48). GR binding to AP-1 motifs occurs through GRE-half sites located inside the AP-1 motif, while GR recognizes cryptic HREs at NF-κB binding sites. This direct GR occupancy blocks the subsequent binding of AP-1 and NF-κB to their binding sites, ultimately restricting inflammatory signaling. Thus, direct DNA binding of GR is likely the most prevalent crosstalk mechanism to modulate inflammatory TF action. Beyond inflammatory TFs, GR can also suppress ER chromatin binding in breast cancer cells (49). Whether this occurs through direct blocking of ER binding or through GR-mediated modulation of chromatin accessibility remains to be investigated.

The initial paradigm posited that GR’s anti-inflammatory effects are mediated by GR monomers through tethering, whereas GR dimers mediate harmful metabolic effects via direct DNA binding (3, 17). Consequently, significant efforts have been devoted to developing dissociated ligands that preferentially induce GR monomers over GR dimers. While many dissociated ligands have been studied, only a few have advanced to clinical trials (10). Notably, given that the GR monomer is nearly nonfunctional (6) and direct DNA binding is the predominant mode of GR action (46–48), the effects of these ligands likely arise from mechanisms other than monomerization and tethering. Intriguingly, GR mutants used in establishing the dissociated ligand paradigm affect GR-mediated recruitment of chromatin remodeling complexes to chromatin (6), suggesting that the coregulator profile induced by these ligands could be the primary determinant of their effects.

The mechanisms mentioned above represent GR’s direct crosstalk pathways aimed at suppressing inflammation. However, GR can also indirectly influence other signaling pathways by generating transcripts that disrupt the activity of other TFs (**Figure 1D**), contributing to its anti-inflammatory effects (50). Anti-inflammatory proteins produced upon GR activation hinder inflammatory signaling by impeding signal transduction and transcription. One notable mediator induced by glucocorticoids is glucocorticoid-induced leucine zipper (GILZ), which directly binds to subunits of inflammatory TFs AP-1 and NF-κB, inhibiting their activity by impeding DNA binding or nuclear translocation (51–53). Besides GILZ, other GR-regulated anti-inflammatory factors have also been implicated in restraining NF-κB activity (54). Through the regulation of these anti-inflammatory factors, GR activation can have long-term effects on inflammation depending on the half-life of these factors.

In addition to influencing inflammatory TFs, GR activation can modulate other signaling pathways through a process termed TF cascade (**Figure 1D**) (55). For example, during fasting, GR activation induces the expression of its target gene, peroxisome proliferator-activated receptor alpha (PPARA), leading to increased protein levels of PPARα, which subsequently regulates its target genes. Thus, GR, through indirect crosstalk mechanisms, facilitates the regulation of PPARα target genes. Intriguingly, the duration of glucocorticoid treatment can also contribute to the TF cascade. In mouse hepatocytes, only chronic glucocorticoid exposure has been shown to induce AR expression (56). The TF cascade mechanism can also occur in reverse fashion, often observed during the development of drug resistance (**Figure 1E**). In the case of GR, its gene expression is restricted by AR, and the inhibition of AR activity by antiandrogens leads to the de-repression of GR gene expression (16, 57). This mechanism is further discussed in the subsequent chapters.

Another indirect crosstalk mechanism is coregulator squelching (**Figure 1F**). Since several TFs utilize the same set of coregulators, one activated TF can sequester coregulators from another TF, leading to the activation of an enhancer at the expense of the other (58). In the case of GR, it has been demonstrated that GR can sequester the EP300 coactivator to its own binding sites from non-GR regulatory loci (33). This squelching facilitates rapid gene repression, which could be attenuated by GR knockdown and rescued by overexpression of EP300. Intriguingly, coregulator squelching could be a prevalent indirect crosstalk mechanism since coactivators have been observed to be rare compared to TFs and corepressors (59).

When exploring beyond the realm of inflammatory TFs, one of the most extensively studied crosstalk mechanisms of GR is pioneering and assisted loading (2). It has been proposed that a specific set of TFs can be categorized as pioneer factors due to their capability to open closed chromatin, thereby facilitating the binding of other TFs such as GR (**Figure 1G**) (60). The ability to bind to and remodel nucleosomes has been suggested as one of the hallmarks of pioneer factor characteristics (61). However, there are diverse ways in which a given TF can interact with a nucleosome (62), suggesting that nucleosome binding ability alone is insufficient to explain the pioneering activity of a TF. Depending on the investigator, pioneer factors can be defined in a variety of ways (63). More recently, it has been suggested that pioneer factors could mediate their effect through a multi-step process that requires passage through cell division to establish open chromatin for other TFs (64). Several TFs have been indicated to act as pioneer factors for GR, including AP-1, CCAAT-enhancer-binding protein beta (CEBPB), myogenic differentiation 1 (MYOD1), and hepatocyte nuclear factor 4A (HNF4A) (27, 65–67). Restricting the activity of these TFs leads to the attenuation of GR chromatin binding.

GR, along with other SRs, can also exhibit pioneering activity, meaning they can initiate chromatin remodeling of closed chromatin (20). This crosstalk mechanism is known as assisted loading, where the initiating TF facilitates the binding of the secondary TF to previously closed chromatin (**Figure 1H**) (68). There are several distinct differences between pioneering and assisted loading (20). First and foremost, assisted loading is context-specific, and the roles of the initiating TF and secondary TF can be reversed depending on the enhancer. Furthermore, the binding events in assisted loading are short-lived, enabling the binding of TFs to the same site without competition (68). In contrast, the pioneer factor model predicts more long-lived binding events (63). Finally, the recruitment of ATP-dependent chromatin remodeling complexes by the initiating TF is crucial in assisting the chromatin binding of the secondary TF, whereas the pioneer factor model traditionally suggests an ATP-independent mechanism (60). Assisted loading was initially characterized for GR, wherein ER chromatin binding was facilitated by GR (68). Subsequently, it was discovered that both ER and GR can assist each other’s chromatin binding (69). In addition to other SRs, GR can also aid the chromatin binding of FOXA1 and cAMP responsive element binding protein (CREB) (26, 55). While reverse assisted loading, facilitated repression (70), has been demonstrated for some nuclear receptors, it is currently unknown if GR possesses such a mechanism.

## 3 Prostate cancer and GR

Prostate adenocarcinomas are predominantly driven by aberrant AR signaling, with the majority of primary prostate cancer tumors being AR-positive and androgen dependent (71, 72). As a result, androgen deprivation therapy (ADT) stands as a main approach for prostate cancer treatment, achieved through procedures like orchiectomy (surgical castration) or treatment with luteinizing hormone-releasing hormone agonists (chemical castration). In advanced stages, ADT, often in combination with chemotherapy, is employed to manage prostate cancer. However, resistance to primary ADT inevitably develops, leading to the emergence of castration-resistant prostate cancer (CRPC). In such cases, second-line antiandrogens like enzalutamide, apalutamide, and darolutamide are utilized to competitively inhibit AR activity (72). These compounds compete with DHT for binding to AR’s ligand-binding domain. Additionally, the activity of AR in CRPC can be suppressed with abiraterone, which blocks the androgen synthesis pathway enzyme CYP17A1. However, abiraterone use reduces serum cortisol levels, which triggers a compensatory rise in adrenocorticotropic hormone and leading to the buildup of mineralocorticoids. To counteract this effect, glucocorticoids are used as an adjuvant treatment to maintain hormonal balance (73).

Despite the administration of second-line antiandrogens, certain CRPCs will inevitably adapt to hormone deprivation and AR signal inhibition (74). This adaptation can occur through various mechanisms that either sustain AR signaling or bypass it altogether. Consequently, CRPC subtypes have been categorized in multiple ways, shedding light on the cancer’s evolving survival strategies (75–77). Recent study classified CRPC into four distinct subtypes based on the chromatin accessibility of enhancers (77). Apart from the subtype with sustained AR signaling (CRPC-AR), three AR-negative subtypes were identified. These subtypes are characterized by WNT signaling (CRPC-WNT), neuroendocrine markers (CRPC-NE), or a stem cell-like phenotype (CRPC-SCL). The latter subtype is also referred to as double-negative prostate cancer (DNPC) due to the absence of AR or neuroendocrine (NE) markers such as chromogranin A (CHGA) and synaptophysin (SYP) (78, 79). AR-negative subtypes are often associated with increased lineage plasticity (80). NE prostate cancer (NEPC) typically arises through trans-differentiation from luminal prostate cells, with its development linked to the loss of tumor suppressor genes PTEN, RB1, and TP53 (75). DNPC has been observed to emerge due to elevated fibroblast or hepatic growth factor signaling (78, 81). The incidence rates of both NEPC and DNPC have notably increased following the introduction of second-line antiandrogens into the drug regimen of patients (78, 82), and NEPC and DNPC exhibit worse survival outcomes compared to AR-positive prostate cancer (79, 83). This underscores the urgent need for new therapeutic targets for AR-negative prostate cancer subtypes.

**Figure 2:**
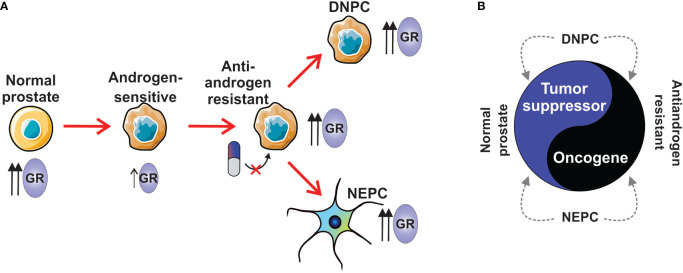
The role of GR in prostate cancer subtypes. **(A)** Normal prostate has higher levels of GR than in androgen-sensitive prostate cancer. During the development of antiandrogen resistance, the expression of GR is increased. AR-negative subtypes, NEPC and DNPC show higher expression levels of GR than androgen-sensitive prostate cancer. **(B)** GR has tumor suppressor role in normal prostate, while it is an oncogene in antiandrogen-resistant prostate cancer. GR’s tumor suppressive or oncogenic role in NEPC and DNPC is unknown. DNPC, double-negative prostate cancer; GR, glucocorticoid receptor; NEPC, neuroendocrine prostate cancer.

The most extensively studied function of GR in prostate cancer is its ability to bypass androgen blockade in AR-positive prostate cancer (16). This phenomenon occurs through the upregulation of GR expression, resulting from the loss of AR-mediated repression, and subsequent replacement of AR signaling. This natural mechanism of acquiring resistance arises from the structural similarity between GR and AR, along with the shared interaction with several coregulators (84). Further insights into this relationship will be provided in the following chapter. More broadly, there are indications suggesting that GR plays a role in various stages of prostate cancer progression (**Figure 2**). In primary prostate cancer, GR expression is significantly decreased or absent compared to normal or benign prostate tissue (15, 85, 86). Moreover, GR expression can be restored to benign levels in metastatic disease (86). Interestingly, different subtypes of CRPC, such as NEPC and DNPC, exhibit higher levels of GR transcripts compared to the AR-positive subtype (RNA-seq data: GSE199190) (77). Taken together, these observations suggest that initially high levels of GR in normal prostate tissue decrease upon the development of androgen-sensitive prostate cancer (**Figure 2A**). Subsequently, GR levels increase in antiandrogen-resistant cancer, as well as in DNPC and NEPC. While GR appears to act as a tumor suppressor in normal prostate tissue (15), it exhibits an oncogenic role in antiandrogen-resistant cancer (16) (**Figure 2B**). Currently, the role of GR in DNPC and NEPC remains largely elusive; however, there are indications of its involvement, which will be addressed in subsequent chapters. It is crucial to recognize that since activated GR possesses strong anti-inflammatory and immunosuppressive effects (31), the use of glucocorticoids can significantly impact the effectiveness of cancer immunotherapy. These challenges and their implications have been extensively reviewed elsewhere (14).

### 3.1 GR crosstalk in AR-positive prostate cancer

The early recognition of the role of glucocorticoids in prostate cancer stemmed from studies involving ligand-binding domain mutated AR (87, 88). These mutations enabled AR to be transcriptionally activated by natural and synthetic glucocorticoids. Subsequently, it was discovered that GR acts as a tumor suppressor in AR-positive prostate cancer, with glucocorticoids inhibiting tumor cell growth by restraining the activity of MAPK signaling (15). The first observed crosstalk partner of GR in prostate cancer was AR (89). Activation of both SRs in prostate cancer cells, with endogenous AR and exogenous GR expression, was found to modulate the transcriptional activity of the other receptor. While this crosstalk bears resemblance to assisted loading (**Figure 1H**), it was not specifically indicated or analyzed in the study.

**Figure 3:**
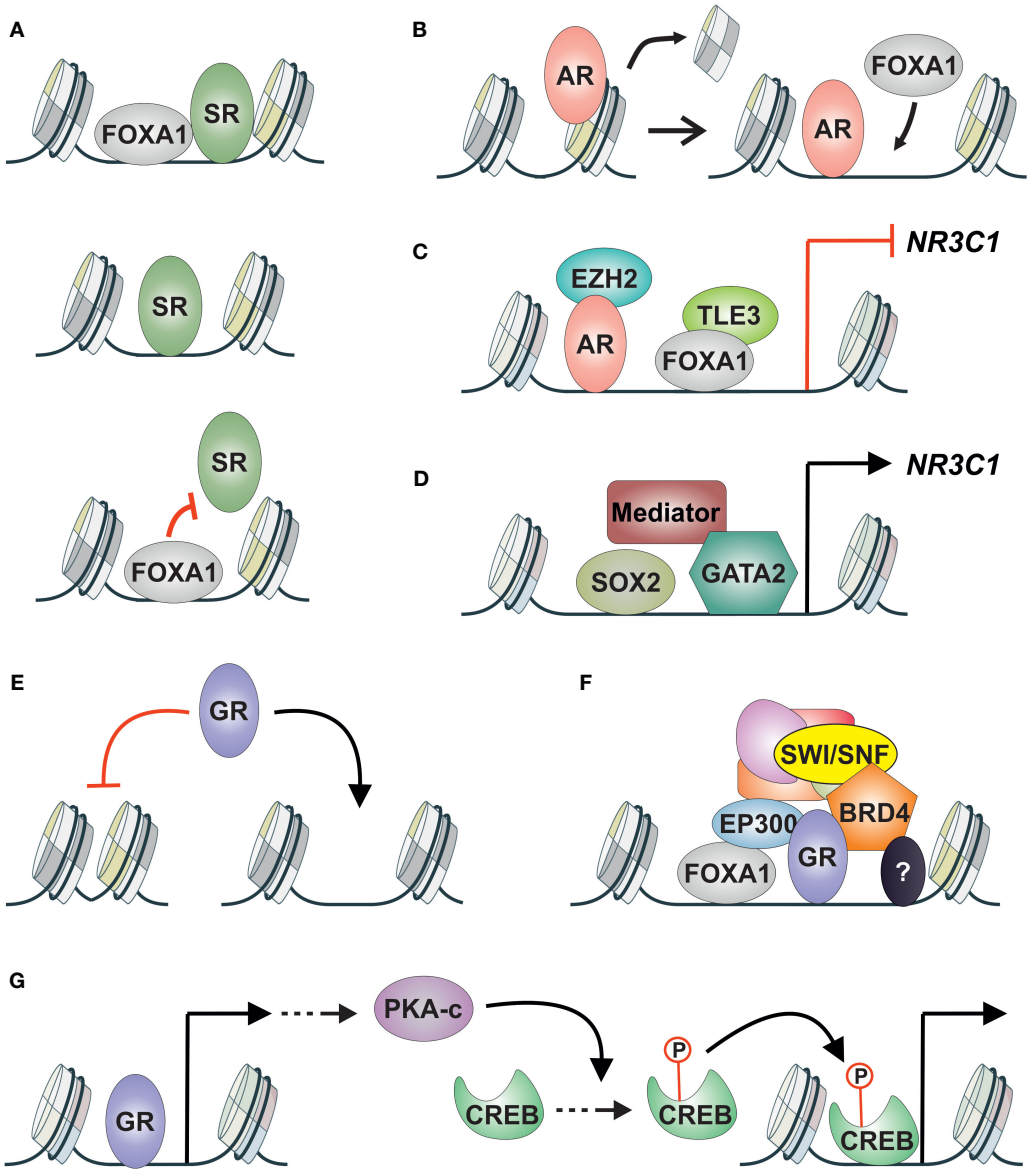
GR crosstalk with other TFs and coregulators in AR-positive prostate cancer. **(A)** SR (GR, AR) crosstalk with FOXA1 can be divided into three categories; (upper) pioneered by FOXA1, (middle) independent of FOXA1, (lower) restricted binding by FOXA1. **(B)** AR can induce the chromatin binding of FOXA1 in prostate cancer cells. **(C)** *NR3C1* expression is repressed by AR via corepressor EZH2, and by FOXA1 via corepressor TLE3. **(D)** *NR3C1* expression is induced by SOX2, GATA2, and the mediator complex. **(E)** GR chromatin binding in AR-positive prostate cancer cells is restricted to open chromatin sites. **(F)** GR-regulated enhancers are associated with FOXA1 and coregulators EP300, BRD4, and SWI/SNF complex. Other TFs are also associated with the enhancers with unknown consequences. **(G)** GR induces the expression of cAMP associated pathway genes leading to nuclear accumulation of PKA-c. PKA-c promotes the phosphorylation of CREB leading to the activation of its transcriptional activity. AR, androgen receptor; CREB, cAMP responsive element binding protein; GR, glucocorticoid receptor; PKA-c, cAMP-dependent protein kinase A catalytic subunit; SR, steroid receptor.

While the binding sites of GR and AR in prostate cancer cells exhibit clear overlap, there seems to be a difference in the composition of enriched motifs: FOXA1 motif enrichment is more prevalent in AR binding sites, whereas ETS family motif enrichment is more prevalent in GR binding sites (89). Both FOXA1 and ETS family proteins are classified as pioneer factors (90). This suggests that AR and GR chromatin binding could be regulated by different pioneer factors (**Figure 1G**). However, despite FOXA1 motif enrichment being more prevalent at AR binding sites, the pioneer factor FOXA1 modulates GR and AR binding in a similar fashion (91). Apart from sites pioneered by FOXA1, several SR-bound enhancers are independent of or restricted by FOXA1 (**Figure 3A**). Hence, FOXA1 does not regulate all GR and AR binding in prostate cancer cells. Furthermore, AR induces the chromatin binding of FOXA1 (**Figure 3B**) (92), although the same has not been demonstrated for GR. Regarding ETS family proteins, ERG is known to modulate the chromatin binding of AR in prostate cancer cells (93). While the relationship between ETS family proteins and GR in prostate cancer is unknown, they directly interact in Ewing sarcoma, influencing the transcriptional activity of GR (94). It is important to note that the specific relationship between GR or AR with certain pioneer factors can depend on the progression state of the cancer cells. AR-positive prostate cancer cells exhibit a distinct chromatin landscape compared to DNPC and NEPC cells (77), suggesting that GR, at least, may interact with different pioneer factors in these various cancer states.

Compared to direct crosstalk mechanisms like assisted loading and pioneering, the indirect interplay between GR and AR has received extensive attention. Patients treated with enzalutamide showed increased levels and transcriptional activity of GR, leading to partial but significant reactivation of AR target gene expression and cancer progression (16). Furthermore, antagonizing GR can mitigate the increased survival rates induced by GR (95). The rise in GR protein levels was attributed to the loss of AR-mediated repression of *NR3C1* (the gene encoding GR) expression via the corepressor EZH2 (85). This AR-GR crosstalk corresponds to the TF cascade mechanism (**Figure 1E**). Interestingly, besides AR, *NR3C1* expression is also regulated by FOXA1 in a similar fashion (57). While AR-mediated repression occurs through the corepressor EZH2, FOXA1 represses *NR3C1* induction via the corepressor TLE3 (**Figure 3C**) (57). Although TLE3’s involvement in repressing *NR3C1* expression had been suggested previously, its link to FOXA1 had remained hidden (96). TF cascade in the opposite direction (**Figure 1D**) has been indicated with GR and SOX2 (97). Depletion of SOX2 leads to decreased levels of GR, suggesting that SOX2 positively regulates *NR3C1* expression. However, there was only a limited effect on the transcriptional activity of GR upon SOX2 depletion, indicating that other TFs contribute to GR signaling. Additionally, GATA2 and the mediator complex (MED1, MED14, MED19) have been implicated in positively regulating *NR3C1* expression (**Figure 3D**) (98, 99). While the roles of FOXA1, SOX2, GATA2, and the mediator complex have not been extensively investigated, AR activity has consistently been associated with decreased GR levels (57, 100). This is supported by the introduction of exogenous AR into the AR-negative cell line, which leads to lowered GR expression levels (100).

Due to the prevalence of indirect crosstalk between AR and GR, combining antiandrogens with GR inhibition presents an appealing strategy to target enzalutamide resistance. Prostate cancer cell models have demonstrated the reversibility of resistance when GR is depleted or its activity is chemically inhibited (16, 95). However, clinical trials involving CRPC patients treated with enzalutamide and the GR antagonist mifepristone or its derivative ORIC-101 did not yield clinical benefits compared to patients treated solely with enzalutamide (18, 19). Despite these outcomes, the utilization of the non-steroidal selective GR modulator relacorilant alongside enzalutamide has shown benefits for a small group of patients in a phase I trial (101). Notably, the patients in the trial had already received several drugs, such as abiraterone, enzalutamide, or chemotherapy, suggesting that less pretreated patients might have derived more benefit from relacorilant. This aspect is likely to be elucidated in subsequent trials. Due to the restricted benefit of relacorilant, alternative pathways should be investigated for their potential to counter aberrant GR signaling. Compared to general GR antagonism, specifically inhibiting GR’s interaction with certain TFs or coregulators could be more advantageous. This targeted approach might suppress GR’s harmful effects while preserving its beneficial actions.

Given that GR binding primarily occurs on pre-accessible chromatin (23), regulators of chromatin accessibility that interact with GR could emerge as new therapeutic targets. This relevance is highlighted by the profound restriction of GR binding sites to open chromatin sites in AR-positive prostate cancer cells (**Figure 3E**) (57), suggesting that GR replaces AR activity and drives enzalutamide resistance from these open chromatin sites. One potential target for regulating chromatin accessibility is FOXA1, which binds to GR binding sites (57, 91). However, since FOXA1 has indirect crosstalk with GR—*i.e.*, FOXA1 represses the expression of *NR3C1*—depletion of FOXA1 does not restrict GR binding at open chromatin sites (57). Instead, GR signaling is potentiated. Because of the dual role of FOXA1—direct crosstalk at GR binding sites and indirect crosstalk through *NR3C1* regulation—alternative approaches to inhibit GR signaling should be explored.

TFs that positively regulate *NR3C1* expression, such as SOX2 (97) and GATA2 (98), could be targeted since they do not pose the same issue as FOXA1 mentioned above. However, there is limited knowledge regarding their interactions with GR at its binding sites. The therapeutic modulation of AR’s transcriptional activity has been achieved through the inhibition of coregulators, such as BRD4, EP300/CREBBP, and the SWI/SNF complex (102–104). Since many coregulators are shared between GR and AR (84), inhibiting coregulator activity could also be used to restrict GR signaling (**Figure 3F**). This is supported by the observation that Bromodomain and Extra-Terminal motif (BET) inhibitors can restrict *NR3C1* expression in enzalutamide-treated prostate cancer cells (85), and GR’s transcriptional activity is modulated in breast cancer cells by depleting SMARCA4, an ATPase subunit of the SWI/SNF complex (105). When comparing BRD4, EP300/CREBBP, and SWI/SNF inhibitors, the most prominent effect on GR signaling is observed with the inhibition of EP300/CREBBP activity (57). Inhibition of EP300/CREBBP’s acetyltransferase disrupts the transcriptional activity of both AR and GR in prostate cancer cells (106). The inhibited AR-regulated transcriptome and chromatin binding are linked to reduced *AR* gene expression, whereas GR’s transcriptome and receptor binding are hindered due to diminished FOXA1 chromatin binding. Although FOXA1 binding is reduced upon inhibition of EP300/CREBBP activity, the repression of *NR3C1* expression by FOXA1 is maintained. Through inhibiting EP300’s acetyltransferase activity, the harmful direct crosstalk between GR and FOXA1 is inhibited while the beneficial indirect crosstalk between the TFs is retained (106). This result underscores the integral role of coregulators in TF crosstalk mechanisms. Indeed, the assisted loading of inflammatory TFs can be curtailed by inhibiting EP300’s enzymatic activity (107). While BET inhibitors, such as JQ1, have also emerged as promising candidates for restricting GR action (85), their usage may pose unexpected risks. JQ1 has been shown to restrict the interaction of FOXA1 with corepressors, such as TLE3 (108), which could lead to increased levels of GR (57). Therefore, a comprehensive understanding of TF crosstalk mechanisms necessitates equal appreciation for coregulators and their inhibitors.

The interplay between GR and FOXA1 suggests that both direct and indirect crosstalk can occur with the same TF pair in the same cellular background. Similar combined crosstalk effects potentially occur between GR and CREB. In enzalutamide-treated prostate cancer cells, GR induces the expression of cAMP pathway-associated genes, leading to the nuclear accumulation of the cAMP-dependent protein kinase A catalytic subunit (PKA-c) (109). This, in turn, results in the phosphorylation (and activation) of CREB, indicating a TF cascade (**Figure 1D**) where GR activation leads to the indirect activation of CREB (**Figure 3G**). While this phenomenon has not been demonstrated in prostate cancer cells, in hepatocytes, CREB can assist the chromatin binding of GR and vice versa (55, 110). Thus, GR and CREB could exist in a feedforward loop in prostate cancer cells, wherein GR indirectly induces the activation of CREB, which in turn leads to the direct modulation of the transcriptional activity of both TFs. Given that CREB signaling can predict resistance to ADT (111), it could represent a significant therapeutic target in GR-mediated antiandrogen-resistant prostate cancer. However, further investigation is required to confirm whether GR and CREB can assist each other’s chromatin binding in prostate cancer cells.

What has not been explored in the described GR crosstalk is the impact of hormone availability and quantity. Direct crosstalk between GR and AR could be influenced by the glucocorticoid-mediated decrease of adrenal androgens (112). Moreover, intra-tumoral glucocorticoid levels increase after enzalutamide treatment due to the decrease of 11β-hydroxysteroid dehydrogenase-2 (11β-HSD2) and the increase of hexose-6-phosphate dehydrogenase (H6PD) levels (113, 114). Ordinarily, 11β-HSD2 inactivates cortisol into cortisone, which is countered by 11β-HSD1. H6PD interacts with 11β-HSD1, generating NADPH for the enzyme to use in the conversion of cortisone to cortisol. Since specific GR-regulated enhancers in lung cancer cells are sensitive to glucocorticoid concentration (115), it is plausible that GR crosstalk, whether direct or indirect, is influenced by hormone concentration. However, further investigation is needed to confirm this hypothesis.

### 3.2 GR crosstalk in AR-negative prostate cancer

While the oncogenic role of GR in enzalutamide-treated AR-positive prostate cancer is well-established (16, 57), its role in AR-negative subtypes has remained elusive. Due to the AR-mediated repression of *NR3C1* expression, AR-negative prostate cancer cells exhibit higher levels of GR compared to AR-positive cells (86). However, the elevated levels of GR in AR-negative cells may not solely be attributed to the absence of AR. These cells also demonstrate low levels of FOXA1, suggesting a deficiency in FOXA1-mediated repression of *NR3C1* (57). Thus, the combined absence of both AR and FOXA1 likely contributes to the heightened levels of GR in AR-negative prostate cancer cells.

Although the explicit role of GR has not been investigated in AR-negative prostate cancer cells, such as NEPC and DNPC, numerous studies have suggested a clear involvement of GR in these subtypes. The significance of GR signaling has been underscored by phenotypical experiments conducted in several AR-negative prostate cancer cell lines. In these experiments, blocking GR activity with a GR inhibitor or depleting GR levels impaired spheroid formation and cell proliferation, emphasizing the essential role of glucocorticoid signaling in the absence of AR (86). Intriguingly, cell lines classified as DNPC cells were utilized in these investigations (77). Additionally, a recent study demonstrated that glucocorticoid treatment increased cell growth of AR-negative prostate cancer cells by altering the secretome of cancer-associated fibroblasts (116). Importantly, these results were not limited to AR-negative subtypes but were also observed in AR-positive prostate cancer cells. This highlights a mechanism of GR-induced cancer cell proliferation mediated by environmental factors, often overlooked in cell culture studies.

**Figure 4:**
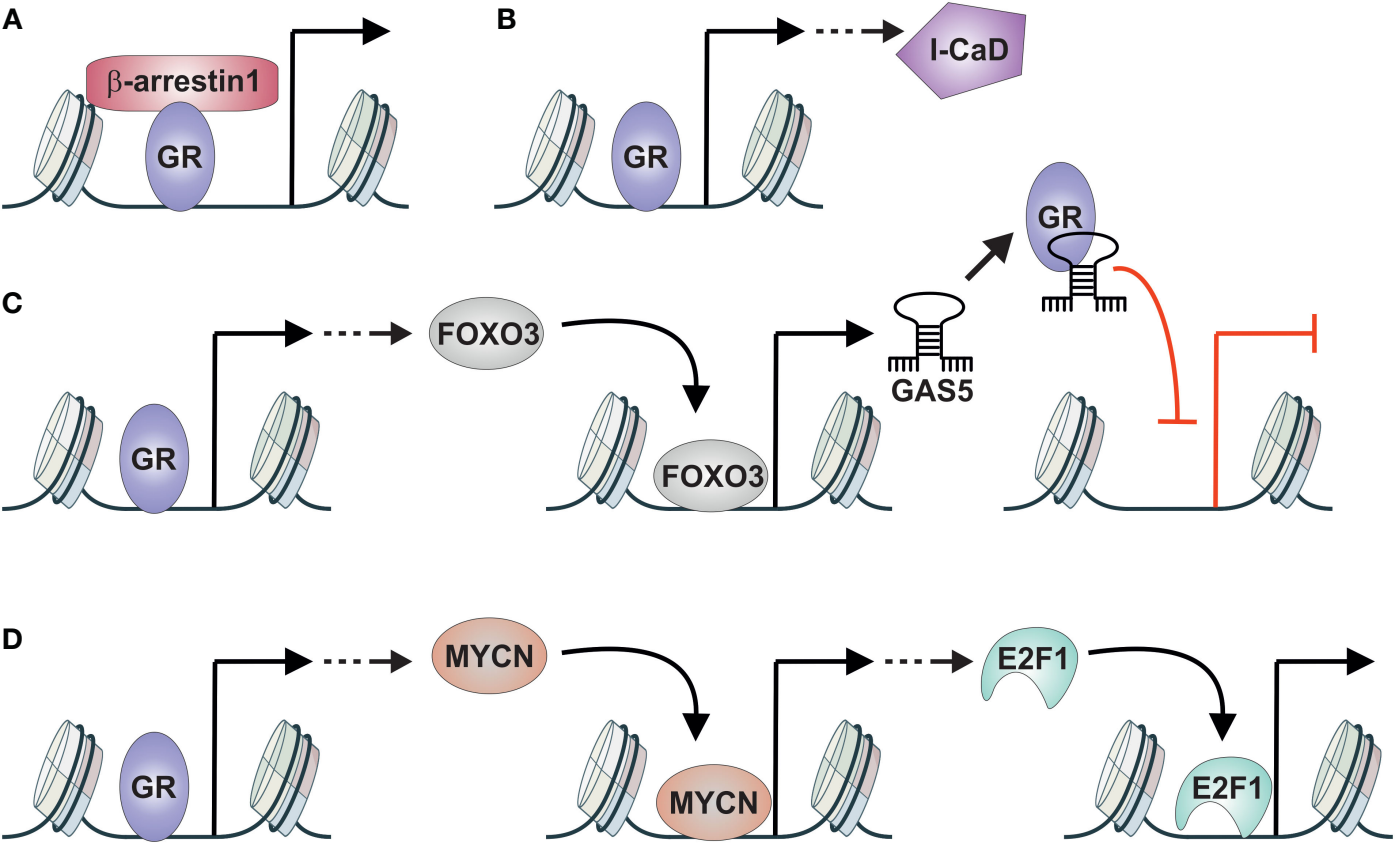
GR crosstalk with other TFs in AR-negative prostate cancer. **(A)** GR interacts with β-arrestin1 to induce spheroid and tumor growth of DNPC cells. **(B)** GR induces the expression of I-CaD that induces spheroid growth of DNPC cells. **(C)** GR induces the expression of FOXO3 that in turn induces the expression of non-coding RNA GAS5. GAS5 acts as GRE decoy repressing the activity of GR in DNPC cells. **(D)** GR induces the expression of MYCN that in turn induces the expression of E2F1. E2F1 promotes the development of NEPC. DNPC, double-negative prostate cancer; GAS5, growth arrest–specific 5; GR, glucocorticoid receptor; I-CaD, low molecular weight caldesmon; NEPC, neuroendocrine prostate cancer.

The above-mentioned studies have highlighted the importance of GR in DNPC cells—others shed light on the potential crosstalk and interacting partners of GR. One such partner is β-arrestin 1, a regulator of G protein-coupled receptor signaling that also exhibits activities in the nucleus (117). β-arrestin 1 was found to interact with GR in the nucleus of DNPC cells, and depletion of it resulted in reduced transcriptional activity of GR and decreased spheroid and tumor growth. Thus, β-arrestin 1 could serve as a crucial coregulator of GR (**Figure 4A**). Another player implicated in glucocorticoid-mediated spheroid growth is low molecular weight caldesmon (l-CaD) (118). I-CaD, associated with microfilaments of migrating cells, suggests its role in the dissemination of cancer cells. Unlike β-arrestin 1, GR does not directly interact with I-CaD but instead regulates its expression (**Figure 4B**). Thus, many of the effects of GR-mediated spheroid formation indicated above could be attributed to the glucocorticoid-induced expression of I-CaD.

Despite the apparent oncogenic role of GR in AR-negative prostate cancer cells, there are indications of the opposite effect. GR activation has been shown to impair AR-negative prostate cancer proliferation through the FOXO3-GAS5 signaling pathway (119). FOXO3 expression is induced by GR, and overexpression of FOXO3 leads to the inhibition of DNPC cell proliferation and migration. Furthermore, GR activation and overexpression of FOXO3 induce the expression of the non-coding RNA growth arrest–specific 5 (GAS5). Intriguingly, GAS5 acts as a decoy GRE, with capability to associate with GR’s DNA-binding domain, thereby impairing the receptor’s functionality (120). This indicates that GR exists in negative feedback loop, where it induces the expression of FOXO3 and GAS5, thereby inhibiting its own activity (**Figure 4C**). This phenomenon is not restricted to the AR-negative subtype, as GR can induce FOXO3 and GAS5 expression in AR-positive prostate cancer cells as well (119, 121). However, FOXO3-GAS5 signaling occurs only in enzalutamide-treated cells, indicating the requirement of high GR levels for their expression. Finally, the crosstalk between GR and FOXO3 represents a TF cascade (**Figure 1D**), as GR activation induces the expression of FOXO3, which acts as a TF on its own.

The results presented above have predominantly focused on GR action in DNPC cells, yet GR also plays a significant role in NEPC. The connection between GR and NEPC occurs once again through a TF cascade (**Figure 1D**) (122). During enzalutamide treatment of AR-positive prostate cancer cells, activated GR induces the expression of MYCN, which subsequently promotes NE differentiation. GR-mediated activation operates through a GRE-containing binding site located in the MYCN promoter. Subsequently, MYCN promotes NEPC through the induction of CDK5 and E2F1 expression (**Figure 4D**). E2F1, in particular, has been implicated in the development of treatment emergent NEPC (123). Moreover, since AR and E2F1 share binding sites in prostate cancer cells and their co-occupancy is dependent on AR (124), a similar interaction between GR and E2F1 could occur in NEPC. Thus, GR could have both indirect and direct crosstalk relationships with E2F1 in NEPC, like the interaction between FOXA1 and GR in AR-positive prostate cancer cells.

Interestingly, GR’s direct crosstalk with other TFs has received relatively little attention compared to the focus on indirect mechanisms discussed earlier. However, there are indications in the literature of potential direct crosstalk partners of GR in DNPC and NEPC. In the characterization of CRPC subtypes, the DNPC (or CRPC-SCL) subtype was defined by several AP-1 subunits, including FOSL1, FOSL2, and JUNB, which were thought to regulate the subtype-specific transcriptome (77). Among these subunits, FOSL1 was ranked the highest. Intriguingly, FOSL1 is associated with the regulation of cancer stemness (125) and can promote the growth and metastasis of prostate cancer cells (126). These findings suggest that AP-1 could be a major transcriptional regulator in DNPC.

Since the direct crosstalk of GR and AP-1 is well known (65), it seems plausible that they would also operate together in DNPC cells. Although GR’s role with AP-1 is best characterized with its JUN subunit, AP-1 hotspots, which indiscriminately recruit multiple AP-1 subunits (including JUN and FOSL1), are responsible for the genomic response of glucocorticoids in lung cancer cells (127). This indicates that FOSL1 likely has a similar crosstalk relationship with GR as JUN does. Intriguingly, the AP-1 subunit FOS exhibits decreased expression during the development of prostate cancer (128), similar to what is observed for GR (85, 86). Conversely, an opposite effect is seen with JUN expression. Since FOS can only form heterodimers with the JUN subunit, whereas JUN can form homodimers (129), the exact composition of AP-1 subunits could significantly influence the activity of GR in the prostate. The loss of FOS could disrupt the equilibrium of AP-1 subunit composition, favoring the formation of JUN-JUN homodimers. This change could potentially modify GR from a tumor suppressor to a more oncogenic transcriptional regulator. Moreover, given that FOSL1 is the prevailing subunit in DNPC (77), the transition from FOS to FOSL1 in the AP-1 complex could also impact the transcriptional activity of GR. Indeed, the composition of JUN and FOS subunits in the AP-1 complex can influence its binding sites in the genome (127). Therefore, alterations in AP-1 subunit composition could affect chromatin accessibility and subsequently impact the chromatin binding of GR. However, whether the altered AP-1 subunit composition directly influences GR signaling requires more detailed investigation.

In addition to AP-1, Janus kinase (JAK)/STAT signaling has the potential to crosstalk with GR in AR-negative prostate cancer. JAK/STAT signaling has been linked to increased plasticity in AR-low prostate cancer, with the potential to progress further to DNPC and NEPC (130). Among the STAT family proteins, GR can directly interact with STAT3 (29), and GR binding to the promoter of STAT1 represses its expression (131). Furthermore, a preprint publication has proposed that ONECUT2 facilitates the development of AR-independent prostate cancer by suppressing AR signaling and facilitating *NR3C1* transcription (132). This suggests an indirect crosstalk between ONECUT2 and GR. Since ONECUT2 drives NE features (133) and its binding sites are enriched with GREs (132), it is likely that direct crosstalk between GR and ONECUT2 exists.

## 4 Future perspectives

While the importance of GR in prostate cancer has been affirmed with the discovery of its capability to replace AR signaling, there are several aspects of GR biology that remain poorly understood in the prostate. Despite the initial decrease in GR expression levels upon cancer initiation and the receptor’s tumor suppressive role (15), the mechanisms underlying the transition of GR from a tumor suppressor to an oncogenic driver are currently unknown. Understanding the mechanisms that drive GR’s tumor suppressive effects in the prostate could potentially be leveraged to counteract the receptor’s oncogenic actions. Additionally, while there is clear evidence that depletion of GR has beneficial effects in AR-negative prostate cancer (86), the overall role of GR in DNPC and NEPC is largely elusive. Moreover, there is conflicting evidence indicating that activated GR can reduce the proliferation of AR-negative prostate cancer cells (119). Since DNPC and NEPC have the worst life expectancy among all prostate cancer subtypes (79), a more comprehensive characterization of GR’s role in these subtypes is warranted. Finally, while GR expression levels are increased in metastatic prostate cancer (86) and GR-induced I-CaD has been shown to promote metastasis (118), the direct role of GR in the formation and progression of prostate cancer metastases remains largely unexplored. We believe that deciphering these unknown aspects of GR biology can be achieved through investigating GR’s crosstalk mechanisms. Additionally, understanding these mechanisms can help identify new therapeutic targets for prostate cancer treatment.

In addition to the TFs that have been investigated, there are various other factors that may modulate GR action in prostate cancer cells. GR binding sites in AR-positive prostate cancer cells are enriched with TF motifs beyond FOXA1, including HOXB13 and members of the ETS family (57, 89), which both play roles in regulating AR action (93, 134). Chromatin proteomic approaches represent a powerful means to discover new interacting partners for a given TF. This methodology has been successfully employed to identify interacting partners for GR in other cellular contexts (135, 136), as well as for AR in prostate cancer cells (137, 138). Chromatin proteomics is likely to uncover novel interacting partners of GR in enzalutamide-treated AR-positive prostate cancer cells, as well as in DNPC and NEPC cells. Furthermore, this approach could strengthen our understanding of the importance of known interacting partners of GR.

GR itself presents intriguing aspects that can influence its action in prostate cancer cells. Rather than a single form, GR exists in multiple isoforms generated through alternative splicing and translation initiation (139). These isoforms can exert substantially different effects on the transcriptional regulation mediated by GR. Notably, over a decade ago, it was demonstrated that the GRβ isoform is present in prostate cancer cells (140). GRβ lacks a portion of the ligand-binding domain and is believed to act as a dominant negative regulator of the main GRα isoform. Depletion of GRβ from DNPC cells, led to attenuation of cell proliferation. Thus, the isoforms of GR may play a significant role in different stages of prostate cancer, although this hypothesis requires further verification.

Finally, despite AR’s repression of *NR3C1* expression, both GR and AR are endogenously co-expressed in certain prostate cancer cell lines and patients (86, 141). This co-expression raises the possibility of direct interaction between GR and AR on chromatin in prostate cancer cells, akin to the interaction observed between GR and ER in non-prostate cells (69). If AR indeed facilitates the chromatin binding of GR, it could exert a dual effect on GR action like FOXA1. In conclusion, our review underscores the significance of GR in various stages of prostate cancer and highlights the pivotal role of TF crosstalk in fine-tuning the action of GR in the disease.

## Author contributions

JH: Visualization, Writing – original draft, Writing – review & editing. LH: Visualization, Writing – original draft, Writing – review & editing. VP: Conceptualization, Visualization, Writing – original draft, Writing – review & editing.
